# Preventing transposons from jumping by transposon-derived small RNAs

**DOI:** 10.1093/plphys/kiaf389

**Published:** 2025-09-01

**Authors:** Wei Yang, Ning Zhang

**Affiliations:** Shandong Engineering Research Center of Food Nutrition and Active Health, College of Food Science and Engineering, Shandong Agricultural University, Tai'an, Shandong 271018, China; State Key Laboratory of Wheat Improvement, College of Agronomy, Shandong Agricultural University, Tai′an, Shandong 271018, China; Assistant Features Editor, Plant Physiology, American Society of Plant Biologists

Transposable elements (TEs) can move from one location to another within a genome. While they are ancient and sometimes provide evolutionary benefits, uncontrolled TE activity can cause mutations and genomic instability. Organisms have evolved various sophisticated mechanisms to suppress TEs from jumping, including epigenetic silencing through DNA methylation, histone modifications, and RNA interference (Levin and Moran 2011; Viviani et al. 2021; Liu et al. 2022). In plants, these regulatory systems generally work as broad-spectrum defense mechanisms targeting many different TEs simultaneously. However, despite these layers of epigenetic control, some TEs remain active. One such element is the DNA transposon Tam3 in *Antirrhinum majus* (snapdragon), which exhibits temperature-dependent transposition and causes visually striking variegation patterns in flowers. The Tam3 element is known to transpose more actively at low temperatures (15 °C) than at standard growth temperatures (25 °C) (Fujino et al. 2011; Zhou et al. 2017). Previous genetic studies identified 2 loci, *Old Stabiliser* (*OSt*) and *New Stabiliser* (*NSt*), that specifically suppress Tam3 activity (Uchiyama et al. 2008). However, the molecular identity of *OSt* and *NSt*, and the exact mechanisms by which they repress Tam3, remained unknown.

Recently, in *Plant Physiology*, Wang et al. (2025) elucidated the molecular mechanisms by which *OSt* and *NSt* inhibit Tam3 transposition. *OSt* is a structurally rearranged, nonfunctional (pseudo) Tam3 element located on chromosome 5, while *NSt* consists of 2 Tam3 elements inserted in an inverted (head-to-head) orientation within the third intron of the transcription factor gene *BREVIS RADIX*. Despite these differences, both loci exert similar suppressive effects on Tam3 transposition without disrupting the core function of the transposase enzyme. Indeed, Tam3 transposase (TPase) transcripts and proteins are produced at normal levels across wild-type, *OSt*, and *NSt* backgrounds, and the enzyme successfully enters the nucleus under low temperature conditions (15 ℃) that typically promote Tam3 transposition. Furthermore, TPase retains its DNA-binding capability in all genetic backgrounds, indicating that the observed suppression by *OSt* and *NSt* does not involve direct interference with the TPase protein itself.

Instead, the authors found that both *OSt* and *NSt* trigger the accumulation of small RNAs (sRNAs) specifically targeting the 5′ terminal region of Tam3, where transposase binding motifs reside (Wang et al. 2025). In *OSt* plants, these sRNAs are mainly 24 nucleotides (nts) in length, while in *NSt* they are predominantly 21 to 22 nts. Functional experiments using virus-induced gene silencing confirmed that artificially induced sRNAs from the Tam3 5′ end were sufficient to suppress Tam3 transposition in *ost*/*nst* plants. Mechanistically, *OSt* and *NSt* exhibit markedly different molecular architectures and transcriptional strategies. *OSt* harbors a pseudo-Tam3 element that underwent a rearrangement at its 5′ terminal region. This rearranged structure allows for bidirectional transcription, generating overlapping sense and antisense transcripts that can anneal to form double-stranded RNAs, which are subsequently processed predominantly by Dicer-like 3 (DCL3) into 24-nt sRNAs. 24-nt sRNAs are typically associated with transcriptional silencing pathways (Erdmann and Picard 2020; Kryovrysanaki et al. 2022). In contrast, *NSt* contains 2 Tam3 elements arranged in an inverted, head-to-head orientation. Transcription from a promoter upstream of the left Tam3 element produces a unidirectional transcript spanning both elements, folding back on itself to form a hairpin RNA structure. This hairpin is cleaved primarily by DCL1 and DCL2, yielding 21- to 22-nt sRNAs, which are generally associated with post-transcriptional silencing (Erdmann and Picard 2020; Kryovrysanaki et al. 2022). Despite these differences in origin and processing, both loci produce specific sRNAs derived from their own 5′ terminal regions containing multiple TPase binding motifs.

This study reveals that the *OSt* and *NSt* loci are transposon-derived repressors that suppress Tam3 transposition by producing sRNAs that interact with the TPase binding motifs within the Tam3 element or with the TPase itself (Fig. 1). Unlike conventional epigenetic silencing, the repression mediated by *OSt* and *NSt* appears to operate independently of transcriptional repression or protein inhibition, pointing to a novel layer of genome regulation. However, several questions remain: Do these small RNAs directly block transposase binding, or do they act through other molecular intermediaries, such as DNA methylation of TPase binding motifs, that could reduce binding affinity or cleavage efficiency? How widespread are such TE-specific repressors in plants, and can this mechanism be leveraged in crop biotechnology? Furthermore, what underlies the different strengths of *OSt* (semi-dominant) and *NSt* (dominant) differences in RNA processing or chromatin context? Altogether, this work suggests that transposons may not only be passive genomic elements under host surveillance but that sequence derivatives originating from them can, in some cases, evolve into loci that repress the mobility of related elements. It expands our view of transposon–host coevolution and introduces new possibilities for harnessing these dynamics in plant research and breeding.

**Figure 1.**
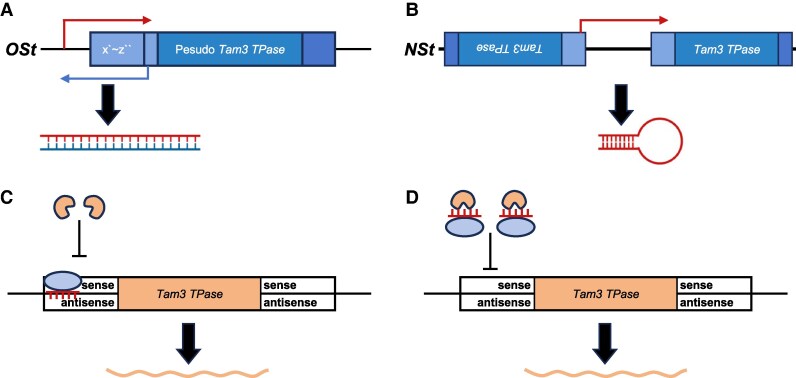
A schematic illustration of *OSt* and *NSt* suppressing the transposition of Tam3 (summarized from Wang et al. 2025). **A** and **B)** Transcription from the *OSt* and *NSt* can generate double-stranded RNAs, which are subsequently processed into sRNAs. Bidirectional transcription of the *OSt* DNA fragment generates complementary sense and antisense transcripts, which anneal to form double-stranded RNA **(A)**. Transcription initiates at the 5′ end of Tam3-L and proceeds through to the 5′ end of Tam3-R. The resulting RNA contains complementary sequences at both ends, allowing it to fold into a stem-loop structure, with double-stranded RNA present in the stem region **(B)**. **C** and **D)** Unknown proteins (ellipse) may associate with these sRNAs and transport them into the nucleus, where these sRNAs may bind to the antisense strand of the 5′ end of Tam3 DNA **(C)** or interact directly with the Tam3 transposase **(D)**, thereby preventing transposase binding to Tam3 DNA and ultimately suppressing Tam3 transposition.

## Data Availability

Data availability statement is not applicable.
